# Supplementary material for: Detection of an Enterococcus faecium Carrying a Double Copy of the PoxtA Gene from Freshwater River, Italy
Source: Antibiotics (Basel). 2022 Nov 13;11(11):1618. doi: 10.3390/antibiotics11111618 (PMC9686737; doi:10.3390/antibiotics11111618)
Supplement: Supplementary file 1 [file antibiotics-11-01618-s001.zip › Table S1.pdf]

Table S1. Primer pairs used for the search of virulence genes.

Gene	Primer Designation	Primer Sequence (5′-3′)	Product size (bp)
gelE	gelE-1 FW	ACGCATTGCTTTTCCATC	419 bp
	gelE-2 RV	ACCCCGTATCATTGGTTT	
cylB	cylB-1 FW	ATTCCTACCTATGTTCTGTTA	843 bp
	cylB-2 RV	AATAAACTCTTCTTTTCCAAC	
cylA	cylA-1 FW	GACTCGGGGATTGATAGGC	688 bp
	cylA-2 RV	GCTGCTAAAGCTGCGCTTAC	
cylM	cylM-1 FW	CTGATGGAAAGAAGATAGTAT	742 bp
	cylM-1 RV	TGAGTTGGTCTGATTACATTT	
esp	esp-1 FW	TTGCTAATGCTAGTCCACGACC	932 bp
	esp-2 RV	GCGTCAACACTTGCATTGCCGA	
ace	ace-1 FW	GAGCAAAAGTTCAATCGTTGAC	1083 bp
	ace-2 RV	GTCTGTCTTTTCACTTGTTTCT	
prgB			
asa1	agg-1 FW	AAGAAAAAGAAGTAGACCAAC	1555 bp
ash701	agg-2 RV	AAACGGCAAGACAAGTAAATA	
