# Supplementary material for: Detection of an Enterococcus faecium Carrying a Double Copy of the PoxtA Gene from Freshwater River, Italy
Source: Antibiotics (Basel). 2022 Nov 13;11(11):1618. doi: 10.3390/antibiotics11111618 (PMC9686737; doi:10.3390/antibiotics11111618)
Supplement: Supplementary file 1 [file antibiotics-11-01618-s001.zip › Table S2.pdf]

SUPPORTING INFORMATION

Table S2 Amino acid sequence identities/similarities of putative proteins encoded by the pEfM1 (GenBank accession no. ON009374).

ORF	Start (bp)	Stop (bp)	Size (amino acids)	Predicted function	BLASTP analysis[a]		
					Most significant database match	Accession no.	% Amino acid identity (% amino acid similarity)
orf1	69	755	228	IS6 family transposase	IS1216 transposase [*Enterococcus faecalis*]	QRN45555.1	100 (100)
Δorf2	1160	735	141	Truncated replication protein	Truncated replication protein [*Enterococcus faecium*]	QRN45573.1	100 (100)
orf3	2250	1384	288	Plasmid recombination enzyme	Plasmid recombination protein [*Enterococcus faecalis*]	HAP3873814.1	100 (100)
orf4	2646	2350	98	Plasmid recombination enzyme	Plasmid recombination protein [*Enterococcus faecalis*]	EHN4298585.1	100 (100)
orf5	4586	3210	458	Tetracycline resistance	Tetracycline efflux MFS transporter Tet(L) [*Enterococcus faecium*]	HBM5951473.1	99 (100)
orf6	6699	4780	639	Tetracycline resistance	Tetracycline resistance ribosomal protection protein Tet(M) [*Enterococcus faecalis*]	EHQ2602673.1	99 (100)
orf7	8008	7076	310		Conjugal transfer protein [*Enterococcus faecalis*]	WP_187167898.1	99 (99)
orf8	8688	8005	227	Lipoprotein, NLP/P60 family	Lipoprotein,NLP/P60 family [*Enterococcus faecium*]	QIZ23697.1	99 (100)
orf9	8744	9430	228	IS6 family transposase	IS1216 transposase [*Enterococcus faecalis*]	TKN59864.1	99 (100)
orf10	10389	12017	542	ABC-F type ribosomal protection protein PoxtA	ABC-F type ribosomal protection protein [*Enterococcus faecium*]	WP_212481470.1	99 (99)
orf11	12065	12751	228	IS6 family transposase	IS1216 transposase [*Enterococcus faecalis*]	TKN59864.1	99 (100)
orf12	12908	13285	125		Hypothetical protein, partial [*Enterococcus faecium*]	WP_205944253.1	100 (100)
orf13	13398	13679	93	Mobile element protein	Transposase [*Enterococcus faecalis*]	QCJ68387.1	100 (100)
orf14	13889	14101	70		Hypothetical protein BTW26_07690 [*Pediococcus acidilactici*]	APR28895.1	100 (100)
orf15	14790	16199	469	Chloramphenicol/florfenicol efflux protein	Florfenicol exporter protein, FexB [*Enterococcus hirae*]	AEV23029.1	100 (100)
orf16	17863	16529	444		Hypothetical protein [*Enterococcus faecium*]	WP_159373660.1	100 (100)
orf17	17919	18605	228	IS6 family transposase	IS1216 transposase [*Enterococcus faecalis*]	TKN59864.1	99 (100)
orf18	19564	21192	542	ABC-F type ribosomal protection protein PoxtA	ABC-F type ribosomal protection protein [*Enterococcus faecium*]	WP_212481470.1	99 (99)
orf19	21240	21926	228	IS6 family transposase	IS1216 transposase [*Enterococcus faecalis*]	TKN59864.1	99 (100)

orf20	22124	22729	201	Fic domain protein	Fic family protein [*Enterococcus faecium*]	WP_139910168.1	99 (100)
orf21	22745	23317	190	Site-specific recombinase	Recombinase family protein [*Bacteria*]	WP_000170424.1	100 (100)
orf22	24340	23750	196	Integrase, catalytic region	Integrase [*Enterococcus faecium*]	MBK4759323.1	100 (100)
orf23	25523	24837	228	IS6 family transposase	IS1216 transposase [*Enterococcus faecium*]	PQG28801.1	99 (99)
orf24	26020	25745	91	PrgO-like protein	Type III secretion system protein PrgO [*Enterococcus faecium*]	HAQ2772571.1	99 (100)
orf25	26945	25992	317		PrgP [*Enterococcus faecalis*]	AEF32544.1	100 (100)
orf26	27557	29050	497		Primase C-terminal domain-containing protein [*Bacteria*]	WP_000947691.1	100 (100)
orf27	29164	29481	105		Replication control protein PrgN [*Enterococcus faecium*]	ABB46244.1	100 (100)
orf28	29582	30433	283		Hypothetical protein [*Enterococcus faecium*]	HAP7268545.1	99 (100)
orf29	30454	30666	70		Hypothetical protein [*Enterococcus faecium*]	UBL09640.1	100 (100)

[a]For each ORF, only the most significant identity detected is listed
Δtruncated ORFs
